# Does artificial intelligence (AI) boost digital banking user satisfaction? Integration of expectation confirmation model and antecedents of artificial intelligence enabled digital banking

**DOI:** 10.1016/j.heliyon.2023.e18930

**Published:** 2023-08-04

**Authors:** Feras Mi Alnaser, Samar Rahi, Mahmoud Alghizzawi, Abdul Hafaz Ngah

**Affiliations:** a An-Najah National University Palestine, Nablus, Palestine; b Hailey College of Banking and Finance, University of the Punjab, Lahore, Pakistan; c Amman Arab University, Jordan; d Universiti Malaysia Terengganu (UMT), Terengganu, Malaysia

**Keywords:** Artificial intelligence, Consumer expectations, Corporate reputation, Trendiness, Customization, Visual attractiveness, Problem solving, Communication quality, Acceptance of AI enabled banking

## Abstract

In the era disruptive technology the emergence of artificial intelligence has fundamentally improved banking operations. The execution of artificial intelligence is no longer discretionary for financial institutions and now it is considered an essential tool to meet customer expectations. Although artificial intelligence enabled digital banking is faster efficient and effective however user acceptance of digital banking driven by artificial intelligence is in its initial stages. Therefore, current study develops and integrated research framework with expectation confirmation model and examines digital banking user satisfaction and acceptance of AI enabled digital banking. Data were collected from digital banking user through structured questionnaire. Overall, 320 respondents were approached and requested to participate in digital banking survey. In return 251 valid responses were received and analyzed with structural equation modeling. Findings of the structural model indicate that satisfaction is jointly determined by expectation confirmation, perceived performance, trendiness, visual attractiveness, problem solving, customization, communication quality and revealed substantial variance R^2 51.1% in digital banking user satisfaction. Therefore, satisfaction and corporate reputation have shown considerable variance R^2 48.3 in user acceptance of AI enabled digital banking. Moreover, the research framework has revealed substantial predictive power Q^2 0.449 to predict digital banking user satisfaction and Q^2 0.493 user acceptance of artificial intelligence enabled digital banking. Concerning with hypotheses relationships exogenous factors have shown positive and significant impact user satisfaction except trendiness and customization. Practically, this research has suggested that policy makers should pay attention in improving user expectation confirmation, perceived performance, visual attractiveness, communication quality and corporate reputation which in turn enhance satisfaction and boost digital banking user's confidence to accept artificial intelligence enabled digital banking. This study is original as it integrates expectation confirmation model with the antecedents of artificial intelligence and examines user behavior towards acceptance of artificial intelligence enabled digital banking.

## 1 Introduction

The evolution of diverse and disruptive technologies in banking sector has touched heights of digital innovation. For instance in digital banking landscape artificial intelligence enabled digital banking has gained large attention. The modern artificial intelligence driven digital banking services are included facial recognition, conversational bots, voice recognition, machine learning to detect fraud, atomization of cyber security detection, biometrics authentication and humanoid robot [1]. In banking context artificial intelligence enabled banking is defined as application ability to collect data from digital or physical sources, interpret and learn from data and use that data to solve customer queries and complex problems [2,3]. It is established that innovation in digital banking is no longer discretionary and being accepted widely by financial institution to achieve distinct and discern customer experiences [[4], [5], [6], [7]]. Although artificial intelligence is studied in e-health Mahdi, Battineni [8] and education setting however little research is found that talked about artificial intelligence enabled digital banking. Therefore, current study has established an amalgamated model with the determinants of artificial intelligence and expectation confirmation model and investigates digital banking user satisfaction and digital banking driven by artificial intelligence.

The acceptance of artificial intelligence among fin-tech institutions is still in its initial stages [4]. For instance Ransbotham, Kiron [9] revealed that 39% executives have no strategic plan to execute artificial intelligence enabled technologies in their organizations. On the flip side institutions with artificial intelligence have achieved firm overall performance [[10], [11], [12]]. In banking domain Mogaji, Balakrishnan [13] postulated that artificial intelligence could promote customer engagement and therefore theoretical knowledge on this subject must be enhanced. Another study conducted by Omoge, Gala [4] has disclosed that artificial enabled digital banking enhance consumer experience and positively impact consumer buying behavior. In addition to that artificial intelligence in banking has been studied in chatbot adoption context [14]. Although above studies have established basic understanding about artificial intelligence enabled digital banking however these studies lack detail of the artificial intelligence determinants. The current research is unique in this context as it has schematized five core determinants of artificial intelligence which impact digital banking user satisfaction and acceptance of artificial intelligence. This study adds value in information system literature as it integrates expectation confirmation model with artificial intelligence and hence enhance body of knowledge on this subject. The reminder of this paper is followed by literature review, methodology, data analysis, discussion, research contribution and conclusion.

## 2 Literature review

### 2.1 Expectation confirmation and user satisfaction

The expectation confirmation theory has been remained popular in measuring customer satisfaction while using internet technology [15,16]. The expectation theory was established by Oliver [17] and evaluates user pre-behavioral expectation confirmation/disconfirmation, performance and satisfaction. According to Eren [16] satisfaction is the foundation of user behavior and could be assessed through user expectation confirmation/disconfirmation and service performance. The term customer expectation is defined as what customers expect and what they will receive in return to avail that service [15,18,19]. Therefore, perceived performance represents to customer perception about service attributes, benefits and outcomes [15,18]. This indicates that users first have expectation confirmation or disconfirmation about artificial intelligence enabled banking. If user expectation towards AI based banking meets with user needs and boosts customer satisfaction. Nevertheless, expectation disconfirmation develops negative behavior towards AI based banking [[20], [21], [22]]. On the flip side positive confirmation boost perceived performance resulting higher user satisfaction towards acceptance of artificial intelligence [23,24]. Prior studies have used expectation confirmation theory as theoretical lens to understand user expectation confirmation/disconfirmation and perceived usefulness towards technology services [16,20,23,25,26]. Therefore, following hypotheses are postulated.

H1 Expectation confirmation positively relates to user satisfaction.

H2 Expectation confirmation positively relates to user perceived performance.

H3 Perceived performance positively relates to user satisfaction.

### 2.2 Artificial intelligence features and user satisfaction

The artificial intelligence features like trendiness, attractiveness and problem solving have made banking operations more attractive, appealing and innovative. For instance author like Chung, Ko [27] stated that customer prefer to use trendy services instead of conventional services. Another dynamic change in business world is that salesperson importance has been reduced and now customers are relying on online system to uplift their life style [28]. Earlier studies have revealed that artificial intelligence based digital banking meet with customers need and their trendy life style which in turn enhance user satisfaction [[27], [28], [29]]. Aside of trendiness digital banking services should be attractive and appealing [[30], [31], [32]]. The term visual attractiveness is seen as individual perception that online interface is colorful, bright, clean, clear, creative, expressive and attract user [33]. In banking context author like Kuo [34] has asserted that visual attractiveness leads to a feeling of arousal and excitement and reduces banking user switching intention. Similarly extensive literature has confirmed that good aesthetic design influence e-user satisfaction and boost customer experiences [30,[35], [36], [37]]. Another important aspect of technology is that it should be problem solving [38]. Therefore, artificial intelligence in digital banking enabled services provider to handle customer problems round the clock resulting higher customer satisfaction [27,38]. Thus, following hypotheses are assumed.

H4 Trendiness of AI positively relates to user satisfaction.

H5 Visual attractiveness of AI positively relates to user satisfaction.

H6 Problem solving of AI positively relates to user satisfaction.

Measuring e-commerce user's need with a single parameter is critical and therefore customization is essential in e-services [39]. The term customization is the degree wherein e-service can be modified, personalized, and adaptable to satisfy customer needs and preferences. According to Perna, Runfola [40] customization in e-service develops strong association between service provider and customers resulting high satisfaction and loyalty towards product. Prior studies have confirmed that artificial intelligence based applications assist customers to avail customized services through chatbot and satisfy their needs [27,41]. Similarly, in digital banking scenario it is found that artificial intelligence enabled banking comprises customization features to assist banking user and enhance digital banking user satisfaction. Communication quality is another essential feature of artificial intelligence based banking. Communication quality is the degree wherein service agent provides accurate, credible, efficient, problem solving and time saving information to customers [41,42]. According to Chakrabarty, Widing [43] rich contents and relevant information reduces uncertainty and enhance customer satisfaction. Moreover, studies have established that e-service agents provide efficient information about product/service, build positive relationship and boost customer satisfaction towards digital banking service [13,43,44]. It is also found that if consumers perceive adequate quality communication through e-service agent, they will enjoy artificial intelligence based digital banking [27,28,44]. In light of above discussion and supported by Mogaji, Balakrishnan [13], Chung, Ko [27], De Andrade and Tumelero [41] following hypotheses are proposed.

H7 Customization feature of AI positively relates to user satisfaction.

H8 Communication quality feature of AI positively relates to user satisfaction.

### 2.3 Corporate reputation

**Fig. 1:**
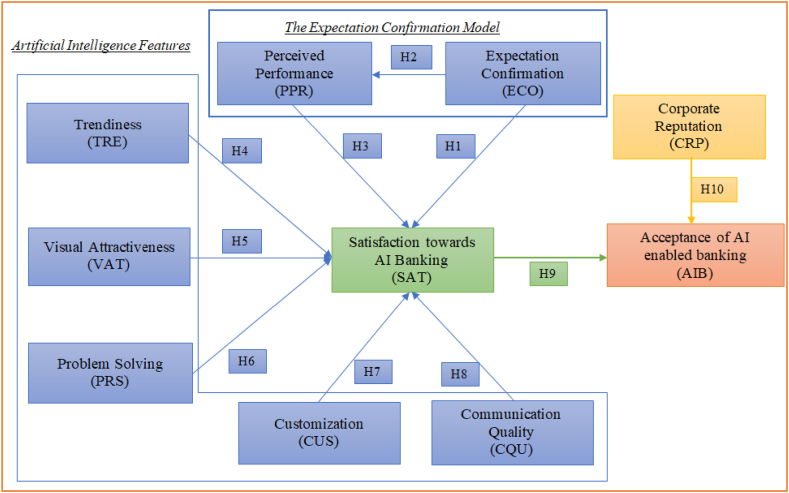
Research framework.

The corporate reputation is an essential element in e-commerce and contributes to firm value. There is strong evidence that corporate reputation positively influence user attitude, reduce anxiety and uncertainty towards digital banking products [45]. Therefore, corporate reputation is outlined research framework as shown in Fig. 1. In this study corporate reputation is defined as user overall evaluation of digital banking service, interaction with stake holders, communication activities, credibility, reliability, trustworthiness and ongoing corporate activities with service provider [45]. Author like Eren [16] has asserted that corporate reputation is a collective representation of a firms services. It is argued that when customers have less information about e-service they rely on corporate reputation [46,47]. Prior studies have confirmed that corporate reputation enhance e-banking user satisfaction boost user confidence [46,47]. In the context of artificial intelligence author like Eren [16] has confirmed that corporate reputation enhance user satisfaction. Therefore, this study extends the body of knowledge and test causal relationship between user satisfaction and acceptance of artificial intelligence based digital banking and hence formulate following hypothesis.

H9 User satisfaction positively relates to user acceptance of artificial intelligence enabled banking.

H10 Corporate reputation positively relates to user acceptance of artificial intelligence enabled banking.

## 3 Research methodology

### 3.1 Instrument development

The research model of this study is empirically investigated through survey questionnaire. Consistent with positivism research paradigm survey questionnaire is designed and comprise respondents demographic characteristics and scale items. In first section of the questionnaires respondents’ age, gender, education and occupation were asked. Therefore, in second part of survey questionnaire scale items were adopted from prior research studies. The current study model comprises ten latent construct that were measured through scale items. Constructs item for expectation confirmation and perceived performance were adopted from Rahi, Othman Mansour [48]. Instrument for trendiness, problem solving and customization were adopted from Lee and Choi [49] and Chung, Ko [27]. Similarly, satisfaction and communication items were adopted from Chung, Ko [27] and Joosten, Bloemer [50]. Next to this visual attractiveness items were adopted from Lee and Pan [33]. Scale for the construct acceptance of artificial intelligence enabled banking were adopted from Rodrigues, Costa [51]. Therefore, corporate reputation items were adopted from Ageeva, Melewar [52]. Concerning with Likert scale extensive literature has established that five-point Likert scale is more favorable when comparing with seven-point Liker scale [[53], [54], [55]]. Therefore, construct items were measured on five-point Likert scale wherein 1 stands for strongly disagree and 5 indicate to strongly agree. Scale items are exhibited in Table 1.

**Table 1:** Measurement model.

Scale items	Loading≥.60	(α) ≥.70	CR ≥ .70	AVE ≥.50
AIB1: Artificial intelligence enabled banking motivate me to use it.	0.889	0.869	0.920	0.793
AIB2: I am willing to use artificial enabled digital banking for online banking transactions.	0.905			
AIB3: The use of artificial intelligence enabled banking gives me feeling of pleasure.	0.877			
CQU1: Artificial intelligence (AI) enabled digital banking provide credible information to user.	0.805	0.763	0.863	0.678
CQU2: Communication using artificial intelligence (AI) enabled digital banking is more productive and useful.	0.827			
CQU3: Artificial intelligence enabled (AI) digital banking save tremendous amount of time.	0.839			
CRP1: I believe banks providing AI enabled digital banking have good reputation.	0.778	0.800	0.868	0.623
CRP2: I believe AI enabled digital banking has good value for money.	0.859			
CRP3: I admire and feel good about banks providing AI enabled digital banking.	0.754			
CRP4: I believe AI enabled digital banking services are credible and fulfill promises.	0.763			
CUS1: This AI enabled digital banking meets with my personal needs.	0.786	0.796	0.867	0.620
CUS2: I feel that customization allows me to transact better when comparing no-customization digital banking applications.	0.816			
CUS3: The AI enabled digital banking offers valuable features that I couldn't find in conventional digital banking.	0.825			
CUS4: The AI enabled digital banking allows me to perform transactions according to my preference.	0.720			
ECO1: My experience with AI enabled digital banking is better than my expectation.	0.765	0.843	0.895	0.681
ECO2: The benefits of AI enabled digital banking are better than my expectation.	0.867			
ECO3: The AI enabled digital banking had better service level than my expectation.	0.845			
ECO4: My expectations towards AI enabled digital banking are confirmed.	0.820			
PPR1: The AI enabled digital banking increase my productivity.	0.853	0.838	0.891	0.672
PPR2: The AI enabled digital banking allows me to complete tasks more quickly.	0.804			
PPR3: With AI enabled digital banking completing financial task are easier to me.	0.799			
PPR4: The use AI enabled digital banking enhances performance.	0.821			
PRS1: I believe that digital banking led by AI has ability to get the job done.	0.858	0.833	0.900	0.749
PRS2: Digital banking led by AI has ability to handle customer complaint directly and immediately.	0.873			
PRS3: The AI enabled digital banking has ability to solve complex problems.	0.867			
SAT1: I am satisfied with AI enabled digital banking services.	0.833	0.765	0.864	0.680
SAT2: The AI enabled digital banking is according to my expectation.	0.852			
SAT3: Overall I am satisfied with digital banking led by artificial intelligence.	0.787			
TRE1: The AI enabled digital banking gives trendy information about digital banking service.	0.895	0.859	0.914	0.779
TRE2: The AI enabled digital banking gives up-to-date information about digital banking service.	0.901			
TRE3: The use of AI enabled digital banking gives latest information about digital banking service.	0.851			
VAT1: Digital banking application led by artificial intelligence is visually appealing.	0.851	0.810	0.887	0.723
VAT2: The interface of AI enabled digital banking application is attractive.	0.889			
VAT3: The user interface of AI enabled digital banking application is professionally designed.	0.809			

### 3.2 Sampling and data collection

This study investigates factors that impact customer intention to accept artificial intelligence in digital banking. Gauging technology user's behavior is a complex phenomenon and hence caution should be paid in selecting relevant respondents [15]. Therefore, in current research setting digital banking user were identified appropriate respondents when comparing with non-digital banking customers. Consequently the population of this study is digital banking users from all commercial and Islamic banks located in Pakistan. The nature of this study is cross sectional and collects data at one point in time. The priori power analysis is employed for sample size computation [53,56,57]. Result as shown in appendix 1 indicate that sample size of 172 respondents was enough for empirical investigation. Nevertheless, earlier studies have argued that sampling error could be reduced if sample size is higher. Consistently, researcher target is to collect maximum responses using survey questionnaires. Data were collected through convenience sampling approach that has substantial support from literature and seems appropriate in business studies [48,53,58]. Overall, 320 respondents were requested to fill digital banking survey questionnaire. Research survey was started in 2nd week of December 2022 and ended in 1st week of January 2023. In research survey participation was voluntarily with promise that personal information will not be revealed. Among 320 respondents 254 respondents have shown interest to participate in digital banking survey. However, questionnaires were further screened out and 3 questionnaires were discarded due to inappropriate and blank answers. Therefore, 251 responses were used in structural model assessment.

## 4 Data analysis

Data were analyzed with structural equation modeling approach. The structural equation modeling is a multivariate data analysis technique that evaluate complex model. Moreover variance based *(VB-SEM)* approach is taken for path estimation. According to Rahi [59] variance based structural equation molding is favorable when research framework is less developed. Therefore, partial least square based *SEM* is taken for data calculation. Data were estimated with *SmartPLS4* software [60].

### 4.1 Structural equation modeling (SEM)

The structural equation modeling is two-stage approach comprising measurement model and structural model [59,61]. In first stage factors reliability, items reliability, convergent and discriminant validity of the measures were established. Table 1 depicts indicators reliability as factor loading is satisfactory i.e. ≥0.60. Likewise, factors reliability is achieved following threshold value ≥ 0.70 of cronbach alpha and composite reliability [59,61]. Convergent validity was confirmed following threshold value of AVE ≥0.50 average variance extracted [57,59]. Thus, measurement model has confirmed factors reliability, items reliability and convergent validity of the measure.

To get accurate estimation it is mandatory that construct should be discriminant and measure distinct concepts. Therefore, discriminant validity is established with cross loading method [56,62]. The cross loading comparison indicate that loadings of the indicators were higher when comparing with other indicators loading and hence confirming discriminant validity. Table 2 exhibits results of the cross loading analysis.

**Table 2:** Cross loading analysis.

Items	AIB	CQU	CRP	CUS	ECO	PPR	PRS	SAT	TRE	VAT
AIB1	0.889	0.500	0.612	0.512	0.531	0.501	0.543	0.510	0.581	0.484
AIB2	0.905	0.410	0.567	0.447	0.496	0.460	0.521	0.456	0.606	0.456
AIB3	0.877	0.473	0.629	0.415	0.497	0.484	0.523	0.486	0.664	0.417
CQU1	0.390	0.805	0.438	0.402	0.456	0.389	0.475	0.433	0.337	0.434
CQU2	0.395	0.827	0.517	0.450	0.486	0.365	0.467	0.446	0.412	0.464
CQU3	0.495	0.839	0.538	0.569	0.518	0.441	0.527	0.473	0.470	0.449
CRP1	0.572	0.462	0.778	0.429	0.453	0.608	0.551	0.504	0.576	0.553
CRP2	0.634	0.581	0.859	0.502	0.539	0.580	0.592	0.531	0.606	0.507
CRP3	0.416	0.437	0.754	0.366	0.429	0.417	0.441	0.467	0.456	0.406
CRP4	0.480	0.409	0.763	0.343	0.408	0.466	0.404	0.484	0.509	0.325
CUS1	0.344	0.443	0.373	0.786	0.435	0.318	0.420	0.366	0.342	0.430
CUS2	0.342	0.393	0.405	0.816	0.363	0.329	0.400	0.326	0.369	0.380
CUS3	0.526	0.518	0.479	0.825	0.453	0.393	0.465	0.424	0.457	0.402
CUS4	0.384	0.451	0.394	0.720	0.470	0.328	0.331	0.342	0.381	0.380
ECO1	0.476	0.520	0.526	0.550	0.765	0.388	0.445	0.448	0.439	0.474
ECO2	0.460	0.482	0.566	0.526	0.867	0.432	0.522	0.509	0.476	0.554
ECO3	0.450	0.488	0.397	0.376	0.845	0.403	0.498	0.482	0.398	0.433
ECO4	0.502	0.468	0.437	0.361	0.820	0.389	0.518	0.501	0.370	0.451
PPR1	0.460	0.428	0.552	0.444	0.419	0.853	0.527	0.489	0.541	0.449
PPR2	0.409	0.367	0.468	0.329	0.300	0.804	0.452	0.387	0.439	0.381
PPR3	0.454	0.392	0.531	0.333	0.440	0.799	0.522	0.485	0.491	0.456
PPR4	0.447	0.395	0.618	0.321	0.420	0.821	0.530	0.477	0.550	0.401
PRS1	0.497	0.523	0.524	0.432	0.502	0.545	0.858	0.525	0.475	0.471
PRS2	0.484	0.546	0.561	0.477	0.512	0.547	0.873	0.497	0.494	0.548
PRS3	0.560	0.479	0.576	0.436	0.548	0.528	0.867	0.536	0.546	0.548
SAT1	0.470	0.438	0.530	0.423	0.536	0.456	0.537	0.833	0.470	0.471
SAT2	0.504	0.489	0.517	0.368	0.483	0.435	0.442	0.852	0.437	0.472
SAT3	0.366	0.427	0.511	0.363	0.431	0.517	0.509	0.787	0.424	0.439
TRE1	0.607	0.393	0.609	0.488	0.429	0.497	0.504	0.492	0.895	0.496
TRE2	0.639	0.475	0.647	0.438	0.510	0.594	0.588	0.520	0.901	0.466
TRE3	0.591	0.447	0.558	0.378	0.405	0.556	0.441	0.400	0.851	0.428
VAT1	0.339	0.460	0.429	0.410	0.509	0.432	0.474	0.448	0.429	0.851
VAT2	0.542	0.519	0.580	0.505	0.529	0.485	0.593	0.556	0.505	0.889
VAT3	0.389	0.399	0.443	0.356	0.434	0.392	0.453	0.401	0.397	0.809

The Heterotrait monotrait ratio analysis is incorporated to confirm discriminant validity of the factors. The HTMT analysis is the latest statistical approach to measure discriminant validity of the factors [57,63,64]. To confirm discriminant validity researcher has followed guidelines provided by Kline [64] and Gold, Malhotra [63]. It is recommended that threshold values of heterotrait monotrait ratios must be ≤ 0.85 or ≤ 0.90 demonstrating adequate discriminant validity of the factors. Results revealed that none of the HTMT value was higher than 0.85 and hence establishing discriminant validity of the factors. Results of the HTMT analysis are shown in Table 3.

**Table 3:** Heterotrait-monotrait ratio analysis.

	AIB	CQU	CRP	CUS	ECO	PPR	PRS	SAT	TRE	VAT
AIB										
CQU	0.633									
CRP	0.795	0.763								
CUS	0.609	0.732	0.645							
ECO	0.668	0.739	0.704	0.669						
PPR	0.631	0.602	0.792	0.530	0.573					
PRS	0.697	0.747	0.770	0.631	0.717	0.741				
SAT	0.663	0.716	0.803	0.593	0.730	0.704	0.753			
TRE	0.801	0.611	0.815	0.591	0.596	0.727	0.683	0.657		
VAT	0.593	0.686	0.692	0.619	0.697	0.619	0.725	0.699	0.622	

Another method to measure discriminant validity is known as fornell and larcker criterion [65]. This method estimates factors discriminant validity through square root of average variance extracted [65]. Results of the PLS algorithm had disclosed that values of AVE were higher than other factors and hence confirming discriminant validity of the constructs. Table 4 depicts findings of the Fornell & Larcker analysis.

**Table 4:** Fornell Larcker criterion.

Items	AIB	CQU	CRP	CUS	ECO	PPR	PRS	SAT	TRE	VAT
AIB	0.890									
CQU	0.520	0.824								
CRP	0.679	0.606	0.789							
CUS	0.515	0.578	0.528	0.788						
ECO	0.571	0.592	0.584	0.548	0.825					
PPR	0.542	0.484	0.666	0.437	0.489	0.820				
PRS	0.595	0.595	0.640	0.517	0.602	0.623	0.866			
SAT	0.544	0.548	0.630	0.467	0.588	0.566	0.600	0.825		
TRE	0.694	0.496	0.688	0.495	0.510	0.621	0.584	0.538	0.883	
VAT	0.508	0.545	0.577	0.506	0.580	0.517	0.603	0.559	0.526	0.850

### 4.2 Predictor-criterion lateral multicollineraity

The multicollinearity issue can lead towards skewness resulting inaccurate or misleading findings [62,66]. Therefore, lateral multicollineraity is assessed with variance inflation factor. According to Rahi, Ghani [62] when measuring predictor and criterion variables the values of variance inflation factor should not be greater than 3.3 [53,67]. Results of the variance inflation factor revealed that VIF values were less than threshold value 3.3 and hence establishing data validity. Findings of the VIF analysis are shown in Table 5.

**Table 5:** Variance inflation factors statistics.

Constructs	Acceptance of AI enabled banking	Perceived performance	Satisfaction towards AI banking
Communication quality			2.025
Corporate reputation	1.658		
Customization			1.788
Expectation confirmation		1.000	2.044
Perceived performance			2.005
Problem solving			2.381
Satisfaction towards AI banking	1.658		
Trendiness			1.983
Visual attractiveness			1.950

### 4.3 Hypotheses testing

Research hypotheses were tested in second stage of SEM namely structural model assessment. Data were bootstrapped with sample of 5000 consistent with prior studies [68,69]. According to Rahi [69] bootstrapping method mitigate data normality issue and must be incorporated in structural model assessment. Results of the structural model had revealed beta values, t-values, standard errors and p-value to accept or reject hypotheses. Results of the hypotheses analysis are exhibited in Table 6.

**Table 6:** Hypotheses testing.

Hypothesis	Path	β -values	STDEV	t-statistics	Significance	Decision
H1	ECO - > SAT	0.204	0.074	2.776	0.003	Accepted
H2	ECO - > PPR	0.489	0.061	7.962	0.000	Accepted
H3	PPR - > SAT	0.178	0.084	2.115	0.018	Accepted
H4	TRE - > SAT	0.099	0.067	1.490	0.070	Rejected
H5	VAT - > SAT	0.133	0.064	2.084	0.020	Accepted
H6	PRS - > SAT	0.148	0.083	1.782	0.039	Accepted
H7	CUS - > SAT	0.013	0.064	0.208	0.418	Rejected
H8	CQU - > SAT	0.123	0.068	1.812	0.036	Accepted
H9	SAT - > AIB	0.194	0.050	3.903	0.000	Accepted
H10	CRP - > AIB	0.556	0.044	12.601	0.000	Accepted
$R^{2}$ Coefficient of determination						
Satisfaction towards AI banking				0.511		
Acceptance of AI enabled banking				0.483		
$Q^{2}$ Predictive relevance of the model						
Satisfaction towards AI banking				0.449		
Acceptance of AI enabled banking				0.493		

The causal relationship among hypotheses is tested through structural model assessment. Results revealed significant impact of expectation confirmation towards user satisfaction and confirmed with β = 0.204 path, STD-error 0.074, t-statistics 2.776 and significance p 0.003 and confirmed H1. Similarly, expectation confirmation has shown positive impact perceived performance and supported by β = 0.489 path, STD-error 0.061, t-statistics 7.962 and significance p 0.000 hence confirming H2. Therefore, the perceived performance has shown positive impact in determining user satisfaction and confirmed H3: β = 0.178 path, STD-error 0.084, t-statistics 2.115 and significance p 0.018. Concerning with artificial intelligence factors results indicate that trendiness had insignificant impact user satisfaction and therefore H4 was rejected β = 0.099 path, STD-error 0.067, t-statistics 1.490 and significance p 0.070. The visual attractiveness has shown significant impact user satisfaction and statistically supported by β = 0.133 path, STD-error 0.064, t-statistics 2.084 and significance p 0.020 thus accepting H5. Next to this problem solving factors of artificial intelligence has confirmed positive impact user satisfaction and supported by H6: β = 0.148 path, STD-error 0.083, t-statistics 1.782 and significance p 0.039. Moving further customization has shown insignificant impact user satisfaction and thus rejected H7: β = 0.013 path, STD-error 0.064, t-statistics 0.208 and significance p 0.418.

Communication is another essential factor of artificial intelligence and has shown positive impact user satisfaction and statistically confirmed by H8: β = 0.123path, STD-error 0.068, t-statistics 1.812and significance p 0.036. User satisfaction has revealed positive impact in determining user acceptance of artificial enabled banking and statistically confirmed by H9: β = 0.194 path, STD-error 0.050, t-statistics 3.903 and significance p 0.000. Similarly, corporate reputation has revealed positive impact user satisfaction and confirmed by H10: β = 0.556 path, STD-error 0.044, t-statistics 12.601 and significance p 0.000. Results of the hypotheses can be seen in Appendix 2. The overall impact of exogenous factors is measured with coefficient of determination. It is found that digital banking user satisfaction is jointly determined by expectation confirmation, perceived performance, trendiness, visual attractiveness, problem solving, customization, communication quality and revealed substantial variance $R^{2}$ 51.1% in digital banking user satisfaction. Therefore, user satisfaction and corporate reputation have shown considerable variance $R^{2}$ 0.483 in user acceptance of AI enabled digital banking. In addition to that predictive power $Q^{2}$ of the research model is assessed. Results had revealed that model has substantial predictive power $Q^{2}$ 0.449 to measure digital banking user satisfaction and $Q^{2}$ 0.493 user acceptance of artificial intelligence enabled digital banking.

### 4.4 Factors effect size analysis

The research model is further scrutinized with effect size analysis $f^{2}$ [59]. According to Rahi [69] f-square values reveal actual impact of factors and therefore must be incorporated in data analysis. The threshold values of $f^{2}$ are considered large at 0.35, medium effect at 0.15 and 0.02 represent to small effect size [59]. Results as depicted in Table 7 indicate that customization has zero effect size this is due the fact that the relationship between customization and user satisfaction was also insignificant. Therefore, all other factors have shown small effect in measuring digital banking user satisfaction. On the flip side corporate reputation has revealed large effect size in measuring user acceptance of artificial enabled digital banking. Within integration the expectation confirmation has shown large effect size in determining perceived performance.

**Table 7:** Effect size $f^{2}$ analysis.

Satisfaction towards artificial intelligence enabled banking		
Factors	$f^{2}$	Effect size
Communication quality	0.015	Small effect
Customization	0.000	No-effect
Expectation confirmation	0.042	Small effect
Perceived performance	0.032	Small effect
Problem solving	0.019	Small effect
Trendiness	0.010	Small effect
Visual attractiveness	0.018	Small effect
Acceptance of artificial enabled banking		
Factors	$f^{2}$	Effect size
Corporate reputation	0.361	Large effect
Satisfaction	0.044	Small effect
Perceived performance		
Factors	$f^{2}$	Effect size
Expectation confirmation	0.314	Large effect

### 4.5 Importance performance analysis

The importance and performance of the outlined factors were established with importance performance matrix. The importance and performance of the factors assist managers and policy makers to understand actual strength of the factors within integrated research framework [59,69]. To calculate IPMA analysis it is mandatory to select an outcome variable. Therefore, research has selected user acceptance of artificial intelligence enabled banking as an outcome variable. Data were estimated using SmartPLS4. Results as depicted in Table 8 have shown that corporate reputation is the most important factor in measuring user acceptance of artificial intelligence enabled banking. Therefore, user satisfaction is the second most important factor in determining user acceptance of artificial intelligence based digital banking. Therefore, the importance of customization is not attractive when comparing corresponding factors like communication quality, expectation confirmation, perceived performance, problem solving, trendiness and visual attractiveness.

**Table 8:** Factors importance and performance.

User acceptance of artificial intelligence enabled banking		
Factors	Importance	Performance
Communication Quality	0.024	66.635
Corporate Reputation	0.556	62.745
Customization	0.003	64.332
Expectation confirmation	0.057	68.103
Perceived performance	0.035	61.028
Problem solving	0.029	62.415
Satisfaction towards AI banking	0.194	65.407
Trendiness	0.019	59.654
Visual attractiveness	0.026	63.126

**Fig. 2:**
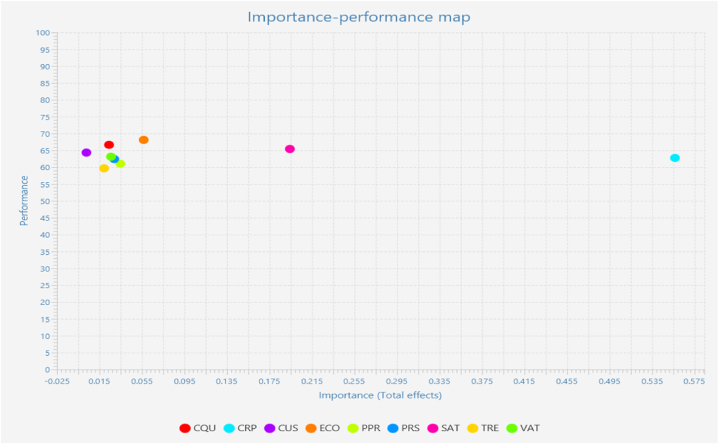
Importance-performance map.

Data were further explored with importance-performance map as given in Fig. 2. The vertical side of the map is showing performance level therefore importance of the factors can be found in horizontal gradient. For managerial point of view factors like corporate reputation, user satisfaction, expectation, problem solving and visual attractiveness are important to be taken into consideration.

## 5 Discussion

In the era of technology advancement and innovative disruption, the artificial intelligence enabled banking has appeared an alternative technology to manage online banking channel, services and solutions. Although advent of artificial intelligence in digital banking has enriched digital banking services however acceptance of AI enabled digital banking and meeting digital banking user expectation are still ongoing issues. Therefore, the present study develops an amalgamated framework to investigate user satisfaction and acceptance of AI enabled digital banking. The research framework has integrated expectation confirmation model with artificial intelligence factors and investigated customer satisfaction towards AI enabled digital banking. Research framework was empirically tested with observation retrieved from digital banking users. The empirical findings of this study have established that expectation confirmation is significantly related to user satisfaction, perceived performance and consistent with prior studies Rahi, Alghizzawi [15], Eren [16]. Similarly, perceived performance has shown positive impact in determining user satisfaction and consistent with Brill, Munoz [23]. Thus, empirical findings have confirmed that digital banking led by artificial intelligence meet customer expectation and boost satisfaction.

Concerning with artificial intelligence factors results indicate that trendiness had insignificant impact user satisfaction and hence rejecting argument developed by Chung, Ko [27]. Therefore, visual attractiveness has shown positive impact user satisfaction and consistent with prior studies Bhandari, Chang [30], Gupta, Gaurav [31], Ho, Le [32]. Moving further problem solving and communication quality have shown positive influence user satisfaction and in line with prior researchers findings [13,43,44]. Contrary to research expectation relationship between customization and satisfaction was found insignificant and hence rejected arguments developed by Perna, Runfola [40]. This is happened because Artificial intelligence is disruptive technology and users had felt difficulty in customization resulting negative feedback. This study has also confirmed that satisfaction and corporate reputation positively impact user behavior to accept digital banking and consistent with Narteh and Braimah [46], Özkan, Süer [47]. Collectively results have shown that expectation confirmation, perceived performance, visual attractiveness, problem solving, communication trendiness and customization explained substantial variance $R^{2}$ 51.1% in digital banking user satisfaction. Likewise, satisfaction and corporate reputation have shown considerable variance $R^{2}$ 0.483 in user acceptance of AI enabled digital banking. The predicative power of the research framework was found substantial $Q^{2}$ 0.449 and $Q^{2}$ 0.493 for satisfaction and user acceptance of artificial intelligence enabled digital banking. In following section research contributions are given in detail.

## 6 Research contribution to theory, method and practice

In terms of contributions this study has several contributions to theory, method and practice. Theoretically, this study has examined impact of expectation confirmation model towards customer satisfaction and acceptance of artificial enabled digital banking and contributes to information system literature. Similarly, this study has confirmed that factors such as visual attractiveness, problem solving and communication quality are essential artificial intelligence factor and enhance user satisfaction and acceptance of AI enabled digital banking. Another theoretical contribution of this study is the integration of expectation confirmation model with artificial intelligence factors. For instance this study integrates factors underpinned expectation confirmation model with visual attractiveness, problem solving, communication quality, corporate reputation, trendiness and customization and investigate user satisfaction and acceptance of artificial intelligence enabled digital banking. Pointing to methodological contribution this study has analyzed data with multivariate data analysis approach namely structural equation Modeling. In addition to that sample size is computed with priori power analysis and hence strengthens research methods. Another contribution to methods is to test research model predictive power with predictive power $Q^{2}$ analysis. Concerning with practical contributions it is found that user expectation confirmation has large effect in determining perceived performance. Therefore, factor like corporate reputation has shown large effect in measuring user acceptance of artificial intelligence enabled banking. In order to reduce model complexity and for clear managerial directions researcher has analyzed data with importance performance matrix. Results revealed that corporate reputation and user satisfaction are key factors which boost digital banking user confidence towards acceptance of artificial intelligence enabled banking. Similarly, the importance of expectation confirmation, perceived performance, visual attractiveness and communication quality was found considerable. These findings clearly indicate that managers and policy makers should pay attention in improving user expectation confirmation, perceived performance, visual attractiveness, communication quality and corporate reputation which in turn enhance user satisfaction and encourage digital banking user to accept and use of artificial intelligence enabled digital banking.

### 6.1 Research limitations and future directions

Although this study has disclosed several useful findings for researcher and practitioners however, it is important acknowledge research limitations for future research directions. First, this study has schematized five core factors of artificial intelligence namely trendiness, customization, communication quality, problem solving and visual attractiveness. Nevertheless, future researcher should not be restricted to these AI factors and hence suggested to add more factors comprising artificial intelligence characteristics. Second, this study has examined user satisfaction and acceptance of artificial intelligence in digital banking. Nonetheless, it is suggested that future researchers should analyze underpinned factors in determining user continuance intention instead of initial acceptance. Third, the research model comprise multiple factors with several assumption and hence complex in nature. Therefore, mediating effect of user satisfaction between user AI acceptance and expectation confirmation was not tested. Thus, future researchers may explore mediating and moderating relationship of artificial intelligence factors and expectation confirmation model towards acceptance of AI enabled digital banking. Fourth, this study is cross sectional and collects user observation at one point in time. Nevertheless, longitudinal research is suggested to reveal interesting findings that how user behavior changes at different stages starting from initial acceptance to continuance use of AI intelligence enabled banking. Finally, this study is conducted in developing country context and therefore replicating current research model in developed region could reveal interesting findings.

## 7 Conclusion

Disruptive technology like artificial intelligence in banking sector is becoming essential to encounter customer expectation and satisfaction. In digital banking landscape artificial intelligence is across the board and could find in facial recognition, conversational bots, voice recognition, machine learning to detect fraud, cyber security detection, biometrics authentication and humanoid robot. Although artificial intelligence enabled banking brings efficiency in data interpretation and have capability to solve customer queries and complex problems the acceptance of AI enabled digital banking is still in its initial stages. Therefore, current study develops and integrated research framework with expectation confirmation model and examines user behavior towards acceptance of AI enabled digital banking. For research design the positivism research paradigm is opted. Data were collected from digital banking user through structured questionnaire. Data were analyzed with structural equation modeling. Results indicate that satisfaction is jointly determined by expectation confirmation, perceived performance, trendiness, visual attractiveness, problem solving, customization, communication quality and revealed substantial variance $R^{2}$ 51.1% in digital banking user satisfaction. Therefore, satisfaction and corporate reputation have shown considerable variance $R^{2}$ 48.3 in user acceptance of AI enabled digital banking. Moreover, the research framework has revealed substantial predictive power $Q^{2}$ 0.449 to predict digital banking user satisfaction and $Q^{2}$ 0.493 user acceptance of artificial intelligence enabled digital banking. Although all exogenous factors have shown positive impact user satisfaction nevertheless the impact of trendiness and customization was found insignificant in determining digital banking user satisfaction. This study contributes to theory and practice in several ways. For instance this study has integrated expectation confirmation model with antecedents of artificial intelligence namely visual attractiveness, customization, problem solving, communication quality and trendiness and examine digital banking user satisfaction. Therefore, integration of expectation confirmation model with antecedents of artificial intelligence contributes to formation system literature. Moving further this research has concluded that managers should pay attention in improving user expectation confirmation, perceived performance, visual attractiveness, communication quality and corporate reputation to boost digital banking user's confidence to accept artificial intelligence enabled digital banking. This study is unique as it has identified core antecedents of artificial intelligence namely trendiness, communication quality, problem solving, customization and visual attractiveness and examine digital banking user satisfaction.

## Author contribution statement

Feras Mi Alnaser: Conceived and designed the experiments; Wrote the paper. Samar Rahi: Conceived and designed the experiments; Performed the experiments. Mahmoud Alghizzawi: Contributed reagents, materials, analysis tools or data. Abdul Hafaz Ngah: Analyzed and interpreted the data.

## Data availability statement

Data will be made available on request.

## Additional information

No additional information is available for this paper.

## Funding information

This research has no funding.

## Declaration of competing interest

The authors declare that they have no known competing financial interests or personal relationships that could have appeared to influence the work reported in this paper.

## Figures and Tables

**Appendix 1:**
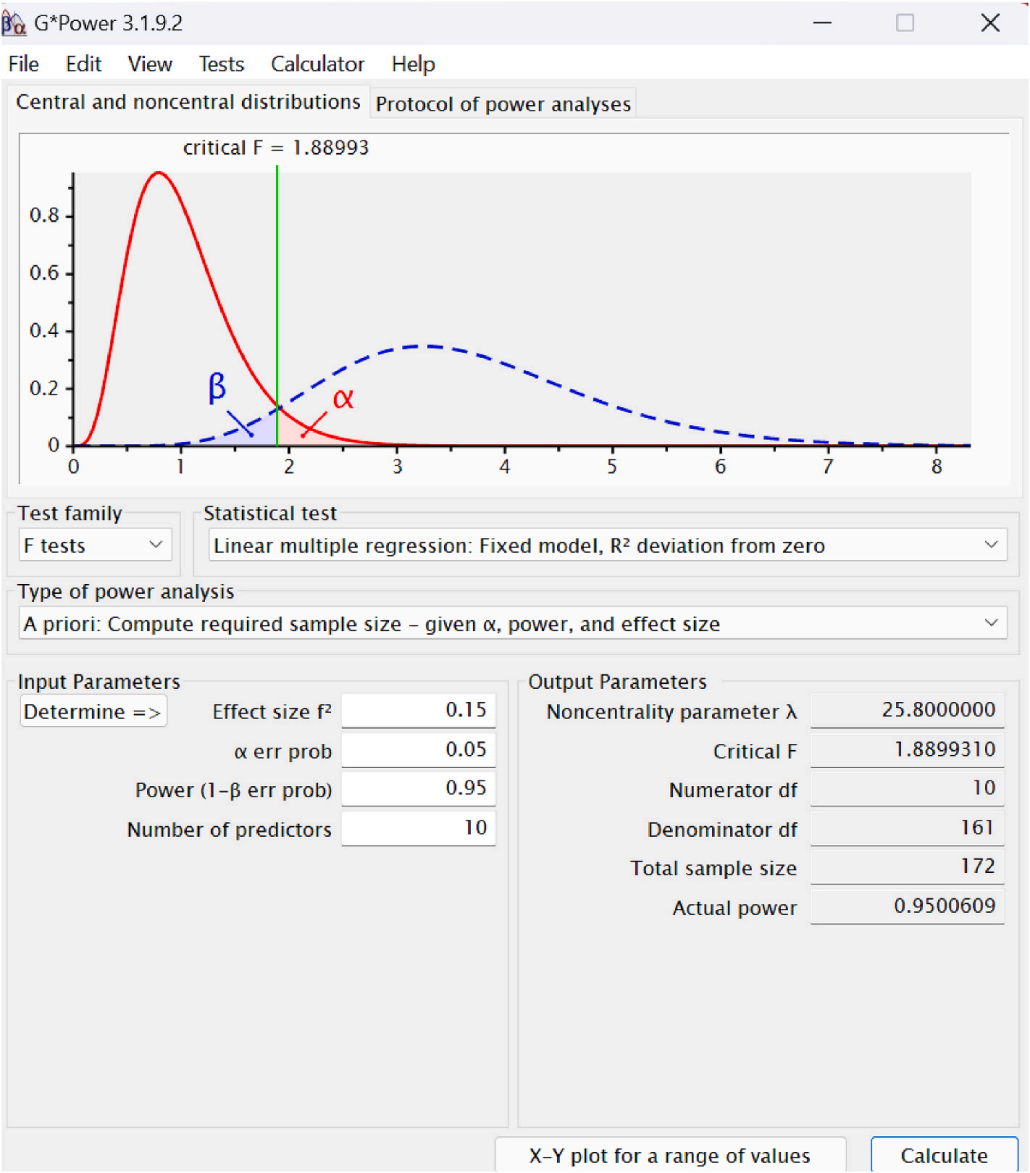
Selecting sample size using Priori Power analysis

**Appendix 2:**
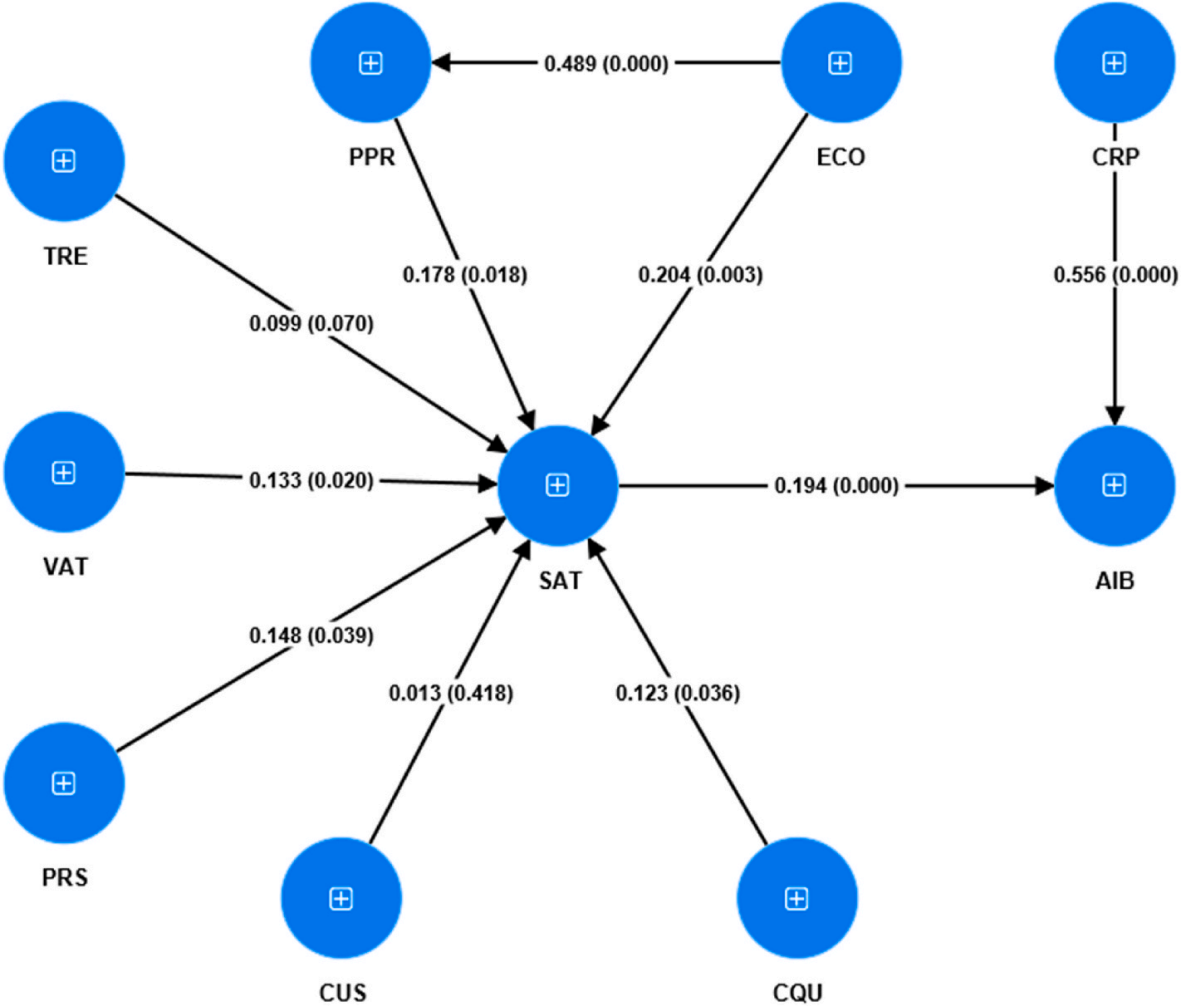
Results of the hypotheses with path and significance level

## References

[1] Al-Okaily M. (2023).

[2] Xu Y. (2020). AI customer service: task complexity, problem-solving ability, and usage intention. Australas. Mark. J..

[3] Kaplan A., Haenlein M., Siri Siri (2019). In my hand: who's the fairest in the land? On the interpretations, illustrations, and implications of artificial intelligence. Bus. Horiz..

[4] Omoge A.P., Gala P., Horky A. (2022). Disruptive technology and AI in the banking industry of an emerging market. Int. J. Bank Market..

[5] Mehrotra A. (2019). 2019 International Conference on Automation, Computational and Technology Management (ICACTM).

[6] Le M.T. (2021). Examining factors that boost intention and loyalty to use Fintech post-COVID-19 lockdown as a new normal behavior. Heliyon.

[7] Al-Qudah A.A. (2022). Mobile payment adoption in the time of the COVID-19 pandemic. Electron. Commer. Res..

[8] Mahdi S.S. (2023). How does artificial intelligence impact digital healthcare initiatives? A review of AI applications in dental healthcare. International Journal of Information Management Data Insights.

[9] Ransbotham S. (2017). Reshaping business with artificial intelligence: closing the gap between ambition and action. MIT Sloan Manag. Rev..

[10] Wamba-Taguimdje S.-L. (2020). Influence of artificial intelligence (AI) on firm performance: the business value of AI-based transformation projects. Bus. Process Manag. J..

[11] Khanagar S.B. (2021). Developments, application, and performance of artificial intelligence in dentistry–A systematic review. Journal of dental sciences.

[12] Al-Okaily M. (2022).

[13] Mogaji E. (2021). Emerging-market consumers' interactions with banking chatbots. Telematics Inf..

[14] Kwangsawad A., Jattamart A. (2022). Overcoming customer innovation resistance to the sustainable adoption of chatbot services: a community-enterprise perspective in Thailand. Journal of Innovation & Knowledge.

[15] Rahi S., Alghizzawi M., Ngah A.H. (2022). Factors influence user's intention to continue use of e-banking during COVID-19 pandemic: the nexus between self-determination and expectation confirmation model. EuroMed J. Bus..

[16] Eren B.A. (2021). Determinants of customer satisfaction in chatbot use: evidence from a banking application in Turkey. Int. J. Bank Market..

[17] Oliver R.L. (1980). A cognitive model of the antecedents and consequences of satisfaction decisions. J. Market. Res..

[18] Venkatesh V. (2003). User acceptance of information technology: toward a unified view1. MIS Q..

[19] Franque F.B., Oliveira T., Tam C. (2021). Understanding the factors of mobile payment continuance intention: empirical test in an African context. Heliyon.

[20] Bhattacherjee A. (2001). Understanding information systems continuance: an expectation-confirmation model. MIS Q..

[21] Azizi M.R. (2021). Innovative human resource management strategies during the COVID-19 pandemic: a systematic narrative review approach. Heliyon.

[22] Bastari A. (2020). Digitalization in banking sector: the role of intrinsic motivation. Heliyon.

[23] Brill T.M., Munoz L., Miller R.J. (2019). Siri, Alexa, and other digital assistants: a study of customer satisfaction with artificial intelligence applications. J. Market. Manag..

[24] Al-Okaily M. (2021). Assessing the effectiveness of accounting information systems in the era of COVID-19 pandemic. VINE Journal of Information and Knowledge Management Systems.

[25] Kim B. (2018). Understanding the role of conscious and automatic mechanisms in social networking services: a longitudinal study. Int. J. Hum. Comput. Interact..

[26] Kosiba J.P. (2020). The moderating role of demographic variables on customer expectations in airport retail patronage intentions of travellers. J. Retailing Consum. Serv..

[27] Chung M. (2020). Chatbot e-service and customer satisfaction regarding luxury brands. J. Bus. Res..

[28] Godey B. (2016). Social media marketing efforts of luxury brands: influence on brand equity and consumer behavior. J. Bus. Res..

[29] Zolkepli I.A., Kamarulzaman Y. (2015). Social media adoption: the role of media needs and innovation characteristics. Comput. Hum. Behav..

[30] Bhandari U., Chang K., Neben T. (2019). Understanding the impact of perceived visual aesthetics on user evaluations: an emotional perspective. Inf. Manag..

[31] Gupta B.B. (2023). Analysis of artificial intelligence-based technologies and approaches on sustainable entrepreneurship. Technol. Forecast. Soc. Change.

[32] Ho M.-T. (2023). Understanding the acceptance of emotional artificial intelligence in Japanese healthcare system: a cross-sectional survey of clinic visitors' attitude. Technol. Soc..

[33] Lee C.T., Pan L.-Y. (2022). Smile to pay: predicting continuous usage intention toward contactless payment services in the post-COVID-19 era. Int. J. Bank Market..

[34] Kuo R.-Z. (2020). Why do people switch mobile payment service platforms? An empirical study in Taiwan. Technol. Soc..

[35] King A.J., Lazard A.J., White S.R. (2020). The influence of visual complexity on initial user impressions: testing the persuasive model of web design. Behav. Inf. Technol..

[36] Wu D., Xu J.D., Abdinnour S. (2021).

[37] Hsieh S.H., Lee C.T., Tseng T.H. (2021). Branded app atmospherics: examining the effect of pleasure–arousal–dominance in brand relationship building. J. Retailing Consum. Serv..

[38] Kim S. (2016). Customer emotions and their triggers in luxury retail: understanding the effects of customer emotions before and after entering a luxury shop. J. Bus. Res..

[39] Bulawa N., Hartwig K. (2023). Serving the Customer.

[40] Perna A. (2018). Problematizing customization and IT in the fashion industry: a case study of an Italian shoemaker. Journal of Global Fashion Marketing.

[41] De Andrade I.M., Tumelero C. (2022).

[42] Yuan T.-T. (2022). Research on the impact of bank competition on stability—empirical evidence from 4631 banks in US. Heliyon.

[43] Chakrabarty S., Widing R.E., Brown G. (2014). Selling behaviours and sales performance: the moderating and mediating effects of interpersonal mentalizing. J. Personal Sell. Sales Manag..

[44] Fares O.H., Butt I., Lee S.H.M. (2022). Utilization of artificial intelligence in the banking sector: a systematic literature review. J. Financ. Serv. Market..

[45] Cintamür İ.G., Yüksel C.A. (2018). Measuring customer based corporate reputation in banking industry. Int. J. Bank Market..

[46] Narteh B., Braimah M. (2020). Corporate reputation and retail bank selection: the moderating role of brand image. Int. J. Retail Distrib. Manag..

[47] Özkan P. (2020). The effect of service quality and customer satisfaction on customer loyalty. Int. J. Bank Market..

[48] Rahi S. (2021). The post-adoption behavior of internet banking users through the eyes of self-determination theory and expectation confirmation model. J. Enterprise Inf. Manag..

[49] Lee S., Choi J. (2017). Enhancing user experience with conversational agent for movie recommendation: effects of self-disclosure and reciprocity. Int. J. Hum. Comput. Stud..

[50] Joosten H., Bloemer J., Hillebrand B. (2016). Is more customer control of services always better?. J. Serv. Manag..

[51] Rodrigues L.F., Costa C.J., Oliveira A. (2017). How does the web game design influence the behavior of e-banking users?. Comput. Hum. Behav..

[52] Ageeva E. (2018). Examining the influence of corporate website favorability on corporate image and corporate reputation: findings from fsQCA. J. Bus. Res..

[53] Rahi S. (2017). Research design and methods: a systematic review of research paradigms, sampling issues and instruments development. Int. J. Econ. Manag. Sci..

[54] Wakita T., Ueshima N., Noguchi H. (2012). Psychological distance between categories in the Likert scale: comparing different numbers of options. Educ. Psychol. Meas..

[55] Hair J.F. (2014). Partial least squares structural equation modeling (PLS-SEM) an emerging tool in business research. Eur. Bus. Rev..

[56] Rahi S., Ghani M., Ngah A. (2018). A structural equation model for evaluating user's intention to adopt internet banking and intention to recommend technology. Accounting.

[57] Rahi S., Alnaser F.M., Abd Ghani M. (2019).

[58] Rowley J. (2014). Designing and using research questionnaires. Management Research Review.

[59] Rahi S. (2017).

[60] Ringle C.M., Wende S., Becker J.-M. (2022).

[61] Hair J.F. (2016).

[62] Rahi S. (2018). Investigating the role of unified theory of acceptance and use of technology (UTAUT) in internet banking adoption context. Management Science Letters.

[63] Gold A.H., Malhotra A., Segars A.H. (2001). Knowledge management: an organizational capabilities perspective. J. Manag. Inf. Syst..

[64] Kline R. (2011).

[65] Fornell C., Larcker D.F. (1981). Structural equation models with unobservable variables and measurement error: algebra and statistics. J. Market. Res..

[66] Kock N., Lynn G. (2012).

[67] Diamantopoulos A., Siguaw J.A. (2006). Formative versus reflective indicators in organizational measure development: a comparison and empirical illustration. Br. J. Manag..

[68] Rahi S. (2022). Does employee readiness to change impact organization change implementation? Empirical evidence from emerging economy. International Journal of Ethics and Systems.

[69] Rahi S. (2022). Assessing individual behavior towards adoption of telemedicine application during COVID-19 pandemic: evidence from emerging market. Libr. Hi Technol..

